# An mHealth-Based Intervention for Adolescents With Type 1 Diabetes and Their Parents: Pilot Feasibility and Efficacy Single-Arm Study

**DOI:** 10.2196/23916

**Published:** 2021-09-14

**Authors:** Bree Holtz, Katharine M Mitchell, Amanda J Holmstrom, Shelia R Cotten, Julie K Dunneback, Jose Jimenez-Vega, Deborah A Ellis, Michael A Wood

**Affiliations:** 1 Department of Advertising and Public Relations Michigan State University East Lansing, MI United States; 2 Department of Communication Michigan State University East Lansing, MI United States; 3 Office of Research Development Clemson University Clemson, SC United States; 4 Sparrow Health System Lansing, MI United States; 5 DeVos Children’s Hospital Grand Rapids, MI United States; 6 Department of Family Medicine Wayne State University Detroit, MI United States; 7 Pediatric Endocrinology University of Michigan Medical School Ann Arbor, MI United States

**Keywords:** mobile health (mHealth), adolescents, type 1 diabetes, mobile phone, parent-adolescent, chronic disease, feasibility, diabetes management

## Abstract

**Background:**

Type 1 diabetes (T1D) affects more than 165,000 individuals younger than 20 years in the United States of America. The transition from parent management to parent-child team management, with the child taking on increased levels of self-care, can be stressful and is associated with a deterioration in self-management behaviors. Therefore, a mobile app intervention, *MyT1DHero,* was designed to facilitate diabetes-specific positive parent-adolescent communication and improve diabetes-related outcomes. The *MyT1DHero* intervention links an adolescent with T1D and their parent through 2 separate app interfaces and is designed to promote positive communication regarding T1D management.

**Objective:**

The aim of this pilot study was to determine (1) the initial efficacy of the *MyT1DHero* intervention in improving diabetes outcomes in adolescents, specifically the hemoglobin A_1c_ (HbA_1c_) levels, diabetes care adherence, and quality of life, and (2) the adolescents’ overall satisfaction with this intervention.

**Methods:**

This pilot study included 30 adolescent-parent pairs who used the *MyT1DHero* app in a 12-week single-arm clinical trial. Participants were recruited from the local pediatric endocrinology subspecialty clinic via snowball sampling. HbA_1c_ levels, diabetes care adherence, quality of life, family conflict, and satisfaction levels were measured and analyzed using paired sample two-sided *t* tests and linear regression analyses.

**Results:**

The final analysis included 25 families. The mean age of the adolescents was 12.28 (SD 1.62) years. Half of the participants (13/25) reported a diabetes diagnosis of less than 5 years. After 12 weeks of the intervention, diabetes care adherence significantly improved (before the study: mean 3.87 [SD 0.59]; after the study: mean 4.19 [SD 0.65]; *t*_21_=–2.52, *P*=.02, *d*=0.52) as did quality of life (before the study: mean 4.02 [SD 0.84]; after the study: mean 4.27 [SD 0.73]; *t*_24_=2.48, *P*=.01, *d=*0*.*32). HbA_1c_ levels (before the study: mean 8.94 [SD 1.46]; after the study: mean 8.87 [SD 1.29]; *t*_24_=0.67, *P*=.51*, d=0.*04) and family conflict (before the study: mean 2.45 [SD 0.55]; after the study: mean 2.61 [SD 0.45]; *t*_23_=0.55, *P*=.14, *d*=0*.*32) changed in the hypothesized direction, but the change was not significant. However, higher use of the mobile app was associated with more improvement in HbA_1c_ levels (*F*_1,20_=9.74, *P*<.005; R^2^=0.33). Overall, the adolescents were satisfied with the app intervention.

**Conclusions:**

In a 12-week pilot study of the mobile app intervention designed to facilitate parent-adolescent communication for improving diabetes outcomes, significant benefits were demonstrated in self-care adherence and quality of life. A randomized controlled trial with a longer intervention is needed to replicate these findings and to determine the stability of the intervention effects.

**Trial Registration:**

ClinicalTrials.gov NCT03436628; https://clinicaltrials.gov/ct2/show/NCT03436628

## Introduction

### Background

Type 1 diabetes (T1D) affects more than 165,000 individuals younger than 20 years in the United States [1,2]. If the child is young at diagnosis, parents initially take over the responsibility for the management of T1D owing to the complexity of the disease and its management to prevent acute and long-term health complications. However, during adolescence, the child begins to take on more responsibility for diabetes self-management. While they are learning the skills needed for self-management, it is vital that parents remain engaged in the process, but they should also prepare their adolescents for independence [3,4].

### Transition of T1D Care

The transition from parent management to a parent-adolescent team management—with the adolescent taking on increased responsibilities of self-care and parents learning to relinquish control—can be stressful and is associated with a deterioration in diabetes self-management adherence behaviors [5]. Diabetes management requires monitoring the blood glucose levels multiple times per day, counting the carbohydrates for meals and snacks, calculating and administering insulin doses by injection or pump, and adjusting the insulin levels or food depending on the glucose readings during a physical activity or illness [6-9]. The transition period, which begins when the child is an adolescent, generally lasts until early adulthood. However, during adolescence, there is often a decrease in the frequency of blood glucose monitoring, an increase in hemoglobin A_1c_ (HbA_1c_) levels, and an increased risk for hospitalization associated with diabetic ketoacidosis. Such deterioration in management can result in poorer glycemic control and potentially lifelong complications [10]. Therefore, it is important to develop interventions to assist in the transition from parent to parent-adolescent team management in order to improve the health-related outcomes for the adolescent.

### Parent and Adolescent Communication

Communication between parents and adolescents is critical at this juncture but is often fraught with difficulty and conflict that is further aggravated by adolescents’ deteriorating adherence to their diabetes management and metabolic control [5,11,12]. Additionally, parents often report feeling worried about their child’s health and feel compelled to check on them frequently [13]. In turn, many adolescents feel that their parents are frequently nagging them about their diabetes care [14]. Reducing parent-adolescent conflict around diabetes management and creating an open and trusting relationship during this tumultuous time is imperative, as a review of the literature found that parent-adolescent diabetes-related conflict is associated with poorer diabetic outcomes [15]. Studies have demonstrated that increased positive reciprocity, problem-solving, and positive communication between parents and adolescents, including optimistic communication, parental social support, and shared decision-making, are associated with positive T1D outcomes [16-19]. Reducing conflict and improving parent engagement during this transition may be possible though the use of technology (ie, mobile phones) to improve communication around diabetes care, thereby improving diabetes outcomes.

### Mobile Health Apps

Mobile health (mHealth) apps have been rated as highly desirable by adolescents with T1D [20]. mHealth enables the use of more engaging strategies such as gamification and customization that have been shown to improve the frequency of blood glucose monitoring in adolescents with T1D [21], and in some cases, improve HbA_1c_ levels when compared to standard care [22,23]. Therefore, an mHealth intervention, *MyT1DHero*, was developed to help support and improve parent-adolescent communication related to diabetes management. The focus of *MyT1DHero*’s is to improve positive communication regarding T1D in an effort to reduce “blood sugar nagging” by parents [24]. These improvements in family communication were, in turn, expected to reduce family conflict, improve adolescents’ adherence to blood glucose monitoring, and thus improve HbA_1c_ levels and quality of life.

### MyT1DHero Intervention

**Figure 1:**
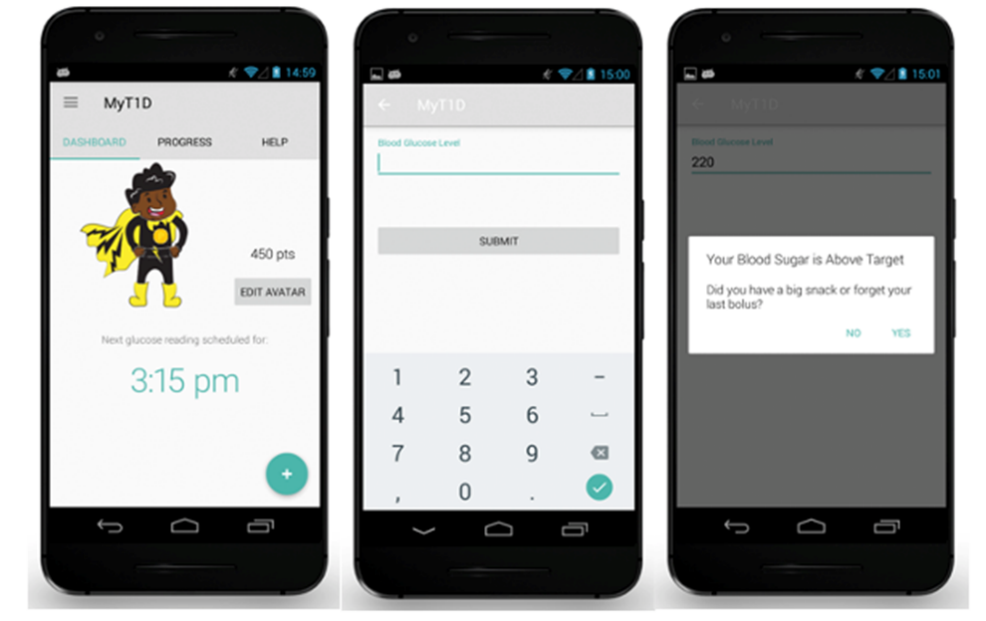
Screenshots of the MyT1DHero app.

The *MyT1DHero* intervention links the adolescent with T1D and their parent through 2 separate app interfaces—one for the adolescent and one for the parent. This app is designed to promote positive communication regarding T1D management. In order to frame the communication in that way, we asked that parents and adolescents establish a blood glucose testing schedule on the app, which will create time-specific reminders for the adolescent to test and enter their blood glucose readings. The app intervention also provides adolescents with tips on how to address out-of-range blood glucose values. For example, when an adolescent enters a low blood glucose reading, the app provides a list of possible snacks (both standard and family-entered). When the child successfully measures and enters the blood glucose values into the app, points are awarded that the adolescents can use to “purchase” accessories for their “hero” avatar on the app. These accessories include capes, boots, logos, masks, and different hair colors and styles. The parents receive notifications about each blood glucose value their child enters. Additionally, parents and adolescents may send each other preprogrammed messages within the app that act as conversation prompts to facilitate positive communication about management, which also earns the adolescent points. In addition to the main functions of the intervention described above, there are also links to videos of other adolescents with T1D telling their stories and providing affirming messages. The reinforcement of diabetes behaviors has been found to help in supporting the adolescents to be more independent, while still keeping the parent aware. See Figure 1 for the screenshots of the app. This intervention is currently only available on an Android platform. Those without a phone or with a phone that was not compatible with *MyT1DHero* were provided a mobile phone for this study. The literature on the development and usability testing of *MyT1DHero* can be found elsewhere [14,25]. The objective of this pilot test was to determine (1) the initial efficacy of the *MyT1DHero* intervention in improving diabetes outcomes, specifically the HbA_1c_ levels, diabetes care adherence, and quality of life, and (2) the adolescents’ overall satisfaction with the *MyT1DHero* intervention.

## Methods

### Study Participants

Eligible adolescents were 10-15 years of age, had a T1D diagnosis for at least 6 months, with HbA_1c_ levels ≥7.0%, fluent in English, had a parent/guardian willing to participate, and allowed to use a mobile phone for this study. Participants were recruited from the local pediatric endocrinology subspecialty clinic through the Southeast Michigan JDRF (formerly Juvenile Diabetes Research Foundation) groups, as well as Facebook, Twitter, word-of-mouth, and snowball sampling. Recruitment to this study took place from September 2017 to December 2017. Thirty adolescent-parent pairs were enrolled in this study. This study was approved by the institutional review board at the Michigan State University and Sparrow Health System.

### Study Design

This pilot study was a single-arm preclinical/postclinical trial without a control group. The intervention was tested over a 12-week period. At the initial visit, informed consent (parent) and assent (adolescent) were obtained. Additionally, venipuncture for determining baseline HbA_1c_ levels was performed at a laboratory (specifically for this study), and pretest survey measures were collected from both the adolescents and the parents.

### Measures

The surveys included questions from the Diabetes Behavior Rating Scale [26], which assesses how well the adolescent is managing diabetes care, and the survey was given to both the adolescent and the parent (see Textbox 1 for example questions, α=.64-.65; all reliabilities reported are based on this study’s sample). This scale has been tested in this population as a measure of adherence several times and includes daily and long-term adherence items [26,27]. This scale has 5 response categories ranging from 1 (Never) to 5 (Always). A higher score indicates a greater level of adherence.

**Survey for the assessment of diabetes care.**

**Examples of questions in the survey for adolescents**

How often…

- was the amount of insulin that your doctor prescribed (including adjustments for diet or blood glucose level) actually taken?
- were your blood sugar numbers written in your log, diary, or chart?
- is insulin correctly adjusted for meals you eat away from home (eg, at restaurants, parties)?
- are clinic or doctor’s appointments kept?
- is your doctor/nurse called for changes in insulin dose if you get frequent “high” or “low” blood sugar levels?
- is your doctor/nurse called if you have severe diabetic symptoms that you cannot correct (eg, drinking a lot, needing fast sugar a lot)?

**Examples of questions in the survey for parents**

How often…

- was the amount of insulin that your child’s doctor prescribed (including adjustments for diet or blood glucose level) actually taken?
- were your child’s blood sugar numbers written in their log, diary, or chart?
- is insulin correctly adjusted for your child’s meals that they eat away from home (eg, at restaurants, parties)?
- are clinic or doctor’s appointments kept?
- is your child’s doctor/nurse called for changes in insulin dose if you get frequent “high” or “low” blood sugar levels?
- is your child’s doctor/nurse called if you have severe diabetic symptoms that you cannot correct (eg, drinking a lot, needing fast sugar a lot)?

Quality of life [28] was self-reported by the adolescent using the PedsQL generic scale (α=.96). This 23-item scale contained 5 response categories ranging from 1 (Never) to 5 (Almost Always); a lower score indicates higher perception of quality of life.

Family conflict was measured using the Revised Diabetes Family Conflict Scale [29] given to both the adolescent and parent. Reliability ranged from α=.94 to α=.95. This 12-item scale has 3 response categories ranging from 1 (Almost Always) to 3 (Never), with a higher score indicating a lower level of conflict.

Satisfaction with the app was measured at the end of the study by using 8 questions from the Poststudy System Usability Questionnaire [30] (α=.84-.85). Satisfaction was measured by asking participants about the different aspects of the app, for example, “I found the *MyT1DHero* app easy to use.” Response options ranged from 1 (Strongly Agree) to 7 (Strongly Disagree), with a lower score indicating higher satisfaction.

Usage was measured in multiple ways. For the number of days used, a day of usage was counted if the child entered the app. The number of days used was grouped as low, medium, and high usage. The low category indicated entering the readings in the app once or less per day for the month, the medium category indicated twice or less per day for the month, and the high category indicated at least 3 times per day. The “high” category indicates proper use of the app as we asked for at least 4 blood sugar entries per day. In addition, the number of blood sugar entries per day and the number of messages sent per day were also considered, and these data were stored by the server. Finally, changes in app usage across the 90-day study period were measured.

### Procedures

At enrollment, participants received a detailed explanation of the *MyT1DHero* app intervention. The parents and adolescents were asked to use the app for 12 weeks, with specific instructions for adolescents to enter their blood glucose readings at least 4 times per day. At the conclusion of the intervention, a repeat venipuncture for HbA_1c_ levels was performed at a laboratory, and posttest surveys were conducted (either online or in-person). Participants were compensated US $50 (US $25 for parents and US $25 for adolescents) after completing the tasks at the initial enrollment meeting. Participants received an additional US $50 (US $25 for parents and US $25 for adolescents) after completing the repeat venipuncture. Finally, an additional US $100 (US $50 for parents and US $50 for adolescents) was given to the participants after completing the posttest surveys and returning phones, if applicable.

### Statistical Analysis

Statistical analyses were performed using SPSS (v.25, IBM Corp). Descriptive statistics were conducted to summarize the sample characteristics and study variables. Paired-sample two-sided *t* tests were conducted to determine if there were statistical differences in HbA_1c_ levels and the other study outcomes from preintervention to postintervention. Additionally, linear regression analysis was conducted to evaluate the association between the adolescents’ usage of the app (high usage, 70-90 days; low usage, 69 days or less) and change in HbA_1c_ levels (pre-HbA_1c_ subtracted from post-HbA_1c_). We also tested the correlations between the adolescent and parent measures, as well as between the age of the adolescent and the satisfaction of the intervention.

## Results

### Participant Characteristics

**Figure 2:**
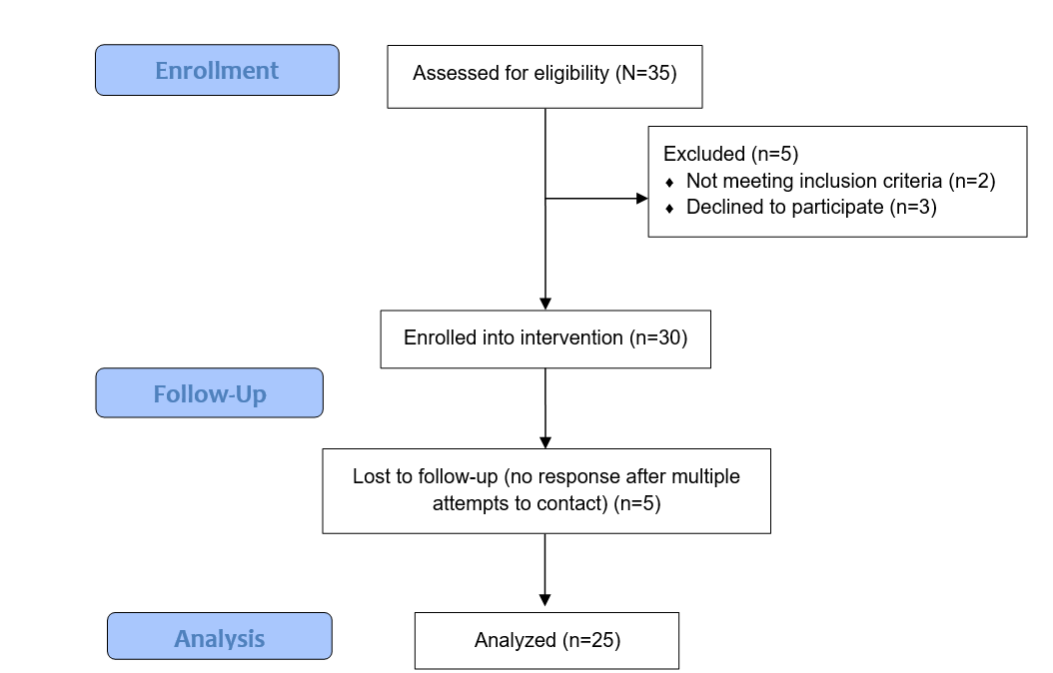
Participant recruitment flowchart.

From September 2017 to December 2017, 30 families were screened and enrolled in this study. The final analysis included 25 families (5 never completed posttest assessments, Figure 2). The majority of the adolescents were Whites (22/25, 88%). The mean age of the adolescents was 12.28 (SD 1.62) years and the majority of the parents was in the age range of 35-44 years (n=10) (see Table 1). Comparing the participants who dropped out, there were no differences in the demographics, except in the length of diagnosis. All the participants who dropped out (n=5) reported that they had been diagnosed for 5 or more years. Of those who completed the study, 12 reported that they had been diagnosed for 5 or more years, 11 had been diagnosed for 1 to 5 years, and 2 had been diagnosed that year (*t*_22_=1.73, *P*<.001).

**Table 1:** Demographics of the participants and outcomes (N=25).

Demographics		Values, n (%)
**Adolescent age (years)**		
	10	4 (16)
	11	4 (16)
	12	7 (28)
	13	5 (20)
	14	1 (4)
	15	4 (16)
**Adolescent gender**		
	Female	12 (48)
	Male	13 (52)
**Length of diagnosis**		
	6 months to less than 1 year	2 (8)
	1 year to less than 5 years	11 (44)
	5 years or more	12 (48)
**Ethnicity/race**		
	White	22 (88)
	Black or African American	2 (8)
	Hispanic or Latino	1 (4)
**Parent relationship to adolescent**		
	Biological mother	22 (88)
	Biological father	1 (4)
	Adoptive mother	1 (4)
	Adoptive father	1 (4)
**Parent age (years)**		
	25-34	1 (4)
	35-44	10 (40)
	45-54	8 (32)
	55-64	3 (12)
**Income (US $)**		
	Less than $25,000	2 (8)
	$25,000-$49,999	4 (16)
	$50,000-$74,999	5 (20)
	$75,000-$99,999	4 (16)
	$100,000 or more	10 (40)
**Insurance**		
	Employer provided	18 (72)
	Medicaid	2 (8)
	Medicare	1 (4)
	Self-insured, non-Medicare, or Medicaid	1 (4)
	Other	3 (12)
**Treatment^a^**		
	Pump	21 (84)
	Continuous glucose monitor	12 (45)
	Multiple daily injections	4 (16)

^a^The participants in this category were on multiple treatments, which is reflected in the percentages in the subcategories.

### Study Outcomes

#### HbA_1c_ levels

After 12 weeks, there were no significant changes (Table 2) in the HbA_1c_ levels (before the study: mean 8.94 [SD 1.46]; after the study: mean 8.87 [SD 1.29]; *t*_24_=0.67, *P*=.51*, d=*0*.*04).

**Table 2:** Results of the outcome variables.

Group, outcome variables		Pretest values, mean (SD)	Posttest values, mean (SD)	*P* value
**Adolescents**				
	Hemoglobin A_1c_ levels	8.94 (1.46)	8.87 (1.29)	.51
	Diabetes behavior rating scale (1=Never to 5=Always)	3.87 (0.59)	4.19 (0.65)	.02
	Quality of life scale (1=Never to 5=Almost Always)	4.02 (0.84)	4.27 (0.73)	.01
	Revised diabetes family conflict scale (1=Almost Always to 3=Never)	2.45 (0.55)	2.61 (0.45)	.14
**Parents**				
	Diabetes behavior rating scale (1=Never to 5=Always)	4.04 (0.50)	4.34 (0.47)	.02
	Revised diabetes family conflict scale (1=Almost Always to 3=Never)	2.47 (0.78)	2.51 (0.85)	.78

#### Diabetes Care Adherence and Quality of Life

Significant improvements were found in the diabetes behavior and quality of life of the adolescents. Posttest measurements (mean 4.19 [SD 0.65]) of diabetes behavior demonstrated improvement compared to the pretest measurements (mean 3.87 [SD 0.59]; *t*_21_=–2.52, *P*=.02, *d=*0*.*52). The parents’ perceptions of diabetes behavior also improved from before the study (mean 4.04 [SD 0.50]) to after the study (mean 4.34 [SD 0.47]; *t*_21_=–2.58, *P*=.02, *d=*0*.*62). Additionally, the scores of the child and the parent Diabetes Behavior Rating Scale were significantly correlated (*r*_24_=0.46, *P*=.04). The quality of life significantly improved after the intervention for the adolescents (mean 4.27 [SD 0.73]) compared to that before the intervention (mean 4.02 [SD 0.84]; *t*_24_=2.48, *P*=.01, *d=*0.32). Although there was an improvement in the family conflict for the adolescents from before the intervention (mean 2.45 [SD 0.55]) to after the intervention (mean 2.61 [SD 0.45]; *t*_23_=0.55, *P*=.14, *d=*0*.*32), the improvement was not significant. The results of the parents’ perception of conflict did not change significantly from before the intervention (mean 2.47 [SD 0.78]) to after the intervention (mean 2.51 [SD 0.85]; *t*_21_=–0.28, *P*=.78, *d=*0.28). The scores of the child and the parent Revised Diabetes Family Conflict Scale were significantly correlated (*r_22_*=0.47, *P*=.03).

#### Satisfaction With the Intervention

Satisfaction with the intervention was measured through 8 questions. Overall, both the adolescents (mean 2.19 [SD 0.94]) and the parents (mean 2.26 [SD 1.29]) rated the intervention as satisfactory. The results of Pearson correlation indicated that there was no association between the age of the child and the satisfaction level (*r*_24_=–0.02, *P*=.92). Each satisfaction item with the descriptive statistics can be found in Table 3.

**Table 3:** Satisfaction of the participants with the MyT1DHero app measured using the poststudy system usability questionnaire.^a^

Variables (scored on a scale of 1-7)	Adolescents		Parents	
	Mean (SD)	Range	Mean (SD)	Range
Overall, I’m satisfied with how easy it is to use this app.	2.70 (1.59)	1-7	2.10 (1.62)	1-7
Learning to use this app was easy.	1.74 (0.94)	1-5	2.00 (1.77)	1-7
The characters on the screen were easy to see.	1.63 (0.79)	1-3	1.48 (0.68)	1-3
The app is understandable.	2.15 (1.13)	1-5	1.97 (1.60)	1-7
It was easy to become skilled using this app.	2.11 (1.25)	1-6	2.00 (1.55)	1-7
I found the app easy to use.	2.30 (1.68)	1-7	1.87 (1.59)	1-7
I think using this app is a good idea.	2.93 (1.94)	1-7	2.19 (1.76)	1-7
Proper type 1 diabetes terms were used throughout the app.	2.04 (1.13)	1-5	2.45 (1.79)	1-7
All satisfaction items	2.19 (0.94)	1-7	2.56 (1.29)	1-7

^a^Response categories: 1=strongly agree, 4=neutral, 7=strongly disagree.

#### App Usage

The average number of days the app was used was 63 (SD 27.70) days. The majority of the participants had usage in the medium or high use categories (21/25, 84%). In addition, participants entered their blood sugar readings an average of less than 3 times per day (mean 2.70 [SD 2.01]) and sent less than 1 message on average per day (mean 0.20 [SD 0.24]) or an average of approximately 23 messages over the course of the entire study period (mean 22.60 [SD 22.38]). Over the 90-day study period, daily use varied. At the start of the study (days 1-5), participants used the app the most times per day (mean 24.54 [SD 9.36]). In the following 60 days, participants used the app on average 7 times per day (mean 7.46 [SD 2.36]). The average use per day then fell further during the final 25 days of the study (mean 4.45 [SD 1.39]). However, linear regression analysis indicated a significant effect between the *overall* use of the app (high/medium/low) and improvement in HbA_1c_ levels (*F*_1,20_=9.74, *P*<.005; R^2^=0.33), demonstrating that the adolescents who used *MyT1DHero* more had greater improvements in their HbA_1c_ levels. We then conducted a separate linear regression to examine if there were any specific functions that predicted the change in HbA_1c_ levels (*F*_5,7_=–5.681, *P*=.21; R^2^=0.80). The data demonstrated that the more the adolescents logged their blood glucose readings and the more messages they sent to their parents, the more their HbA_1c_ levels improved (Table 4).

**Table 4:** Linear regression of the types of usage predicting the change in the hemoglobin A_1c_ levels.

Function	Unstandardized β	SE	β	*t* *(df)*	*P* value
Constant	–1.356	0.332	N/A^a^	–4.089 (24)	.005
Glucose logging (child only)	.004	0.001	1.098	4.798 (24)	.002
Messaging (child)	–.069	0.020	–1.168	–3.492 (24)	.01
Messaging (parent)	.042	0.014	.945	3.024 (24)	.02
Glucose-specific messaging (child)	.066	0.044	.648	1.494 (24)	.18
Glucose-specific messaging (parent)	–.013	0.012	–.470	–1.091 (24)	.31

^a^N/A: not applicable.

Further, participants who used a continuous glucose monitor (CGM) were asked to enter their calibration blood glucose values 1-2 times per day but were allowed to use their CGM number for the other entries. Importantly, there were no significant differences between CGM users and non-CGM users in the usage of the app by category (*t*_24_=0.561, *P*=.58) nor in the days used (*t*_24_=0.092, *P*=.93).

## Discussion

### Overview of This Study

The purpose of this study was to conduct a preliminary evaluation of the efficacy of the *MyT1DHero* mobile app intervention. The goal of the *MyT1DHero* intervention is to facilitate positive diabetes-related communication between adolescents with T1D and their parents to improve diabetes outcomes and help in the transition of diabetes management responsibilities. As the adolescent transitions to more self-care, they need to perform these management behaviors independently, such as monitoring blood glucose levels and taking the appropriate actions if these values are out of range.

### Adherence Improvements in Diabetes Care

The results from this preliminary evaluation demonstrate that diabetes care adherence as reported by both the adolescents and the parents and the quality of life as reported by adolescents improved after using the *MyT1DHero* app. Demonstrating improvement in diabetes management behaviors is critical for this population, especially in this age group, as these behaviors need to be learned and integrated into everyday life for the rest of the adolescents’ life. Studies have demonstrated that perceptions of quality of life significantly affect adherence to medical recommendations and overall health outcomes [30-33]. These findings suggest that participating in this type of intervention could improve primary clinical outcomes such as glycemic control; however, additional studies with longer follow-up periods are needed to see if improvements in diabetes care would be manifested in improved blood glucose levels.

### HbA_1c_ Improvements

Although there was no overall significant effect on the HbA_1c_ levels, the results of our study demonstrate that those who used the app more had more improvements in their HbA_1c_ levels. This indicates the importance of creating an engaging intervention that is tailored to individual families in order to encourage long-term engagement [34]. Moreover, improvements in diabetes behavior and quality of life through long-term use of the *MyT1DHero* app intervention could also positively affect clinical diabetes outcomes; however, further research is needed.

### Adolescent App Satisfaction

This study shows promise for interventions delivered via mobile phones. Past research has shown that mobile phones and apps are perceived by adolescents as a more acceptable way to communicate with a parent, particularly in social contexts in which adolescents may be reluctant to engage in diabetes management, for example, when they are with their peers [35]. Furthermore, while our sample spans 2 developmental stages, this study was conducted to understand the acceptability of the intervention. However, there was no relationship between age and satisfaction of the app intervention, indicating that more qualitative work should be done in this area to determine the acceptability and appropriateness for different ages.

### Communication Between Parents and Adolescents

Future research should include not only assessments of the intervention’s effect on negative communication patterns between parents and adolescents but also the positive interactions. For example, although the use of the intervention did not result in a significant drop in family conflict, it is possible that positive communication behaviors such as self-disclosure, expressions of affection, and social support did increase throughout the length of the intervention. Further, our data demonstrate that when parents initiate the messaging that is perceived to be negative for the child, it can be considered as “nagging” by the parents. However, when the child reached out, there was more positive impact [24]. Future studies should also measure different types of communication—both positive and negative.

### Limitations of This Study

This research has the following limitations. The small sample size was fairly homogenous. The sample reported relatively low levels of baseline family conflict, which may also have limited the study’s ability to show significant reductions in conflict at the 12-week follow-up. This study used self-report measures, which should be noted as a limitation. Providing a mobile phone might have also affected the results. We had to provide phones to participants who had an Apple phone; therefore, these participants ended up carrying 2 phones. Additionally, although we asked families to provide us with the blood glucose trends via a meter download at baseline and at study end, the majority of the families failed to do this. Therefore, an objective measure of blood glucose checking was not available for use in the analyses. Finally, we cannot know for sure if the adolescents entered their blood glucose values accurately and honestly in the app. Further studies are necessary to determine the intervention efficacy in promoting positive communication around T1D management.

### Conclusion

The strengths of this study demonstrate a positive impact on adolescents’ diabetes behaviors and quality of life through the use of an app focused on fostering positive communication with their parents. Mobile phone apps provide a promising avenue for improving parental involvement in the transition of diabetes management from children to adolescents. Using a family-based app intervention provides parents access to their child’s health care treatment and allows adolescents to easily ask for help even when the family is not together. More work regarding the use of similar app interventions is needed to further demonstrate their positive and long-term effectiveness for adolescents with T1D. This study provides preliminary support for the feasibility and efficacy of the *MyT1DHero* intervention for improving health outcomes in adolescents with T1D. mHealth interventions developed to improve the communication between adolescents and their parents during the transition to adolescent self-care could be a promising method to improve health outcomes in this population.

## Abbreviations

CGM: continuous glucose monitor
HbA_1c_: hemoglobin A_1c_
mHealth: mobile health
T1D: type 1 diabetes
